# The *Bdkrb2* gene family provides a novel view of viviparity adaptation in *Sebastes schlegelii*

**DOI:** 10.1186/s12862-021-01774-0

**Published:** 2021-03-17

**Authors:** Jingjing Niu, Weihao Song, Rui Li, Haiyang Yu, Jian Guan, Jie Qi, Yan He

**Affiliations:** MOE Key Laboratory of Marine Genetics and Breeding, College of Marine Life Sciences, Ocean University of China, Qingdao, 266003 China

**Keywords:** *Bdkrb2*, Viviparity, Ovarian wall, Vasodilatation, Adaptation

## Abstract

**Background:**

Black rockfish (*Sebastes schlegelii*) is a viviparous teleost. We proposed that the rockfish ovarian wall had a similar function to the uterus of mammals previously. In the present study, the well-developed vascular system was observed in the ovarian wall and the exterior surface of the egg membrane. In gestation, adaptation of the ovary vasculature to the rising needs of the embryos occurs through both vasodilation and neovascularization. *Bdkrb2*, encoding a receptor for bradykinin, plays a critical role in the control of vasodilatation by regulating nitric oxide production.

**Results:**

Eight *Bdkrb2* genes were identified in the black rockfish genome. These genes were located on chromosome 14, which are arranged in a tandem array, forming a gene cluster spanning 50 kb. Protein structure prediction, phylogenetic analysis, and transcriptome analysis showed that eight *Bdkrb2* genes evolved two kinds of protein structure and three types of tissue expression pattern. Overexpression of two *Bdkrb2* genes in zebrafish indicated a role of them in blood vessel formation or remodeling, which is an important procedure for the viviparous rockfish getting prepared for fertilization and embryos implantation.

**Conclusions:**

Our study characterizes eight *Bdrkb2* genes in the black rockfish, which may contribute to preparation for fertilization and embryo implantation. This research provides a novel view of viviparity adaptation and lays the groundwork for future research into vascular regulation of ovarian tissue in the breeding cycle in black rockfish.

**Supplementary Information:**

The online version contains supplementary material available at 10.1186/s12862-021-01774-0.

## Background

Black rockfish (*Sebastes schlegelii*) is a viviparous teleost, whose embryos develop in the maternal reproductive system from fertilization to birth. Several studies have revealed the viviparity adaptation of black rockfish from the aspects of annual reproductive cycle and sperm storage in the ovary [1–3]. The time course of copulation up to birth spans about 8 months. It takes 6 months for oocytes to mature and fertilize with sperm stored in the ovary [1, 4]. The gestation period is about 50 days [5, 6].Contrast to mammals, where embryonic development occurs in the uterus, embryos develop in the ovary in black rockfish. Black rockfish was traditionally regarded as lechithotrophy viviparity, the embryos of which develop only depending on the yolk from the egg [7]. However, one previous study demonstrated that offspring need to receive nutrition in addition to that supplied in the yolk and uptake of nitrogenous substance occurs through ingestion and absorption of ovarian fluid in the hindgut [5]. The structures in the ovarian system to supply nutrients to the developing embryos have not been characterized so far.

In mammals, mother shares her bloodstream with the fetus via the placenta to exchange nutrients, metabolites and gas. During the whole period of gestation, nutrition is transmitted by the blood circulation between mother and fetus. The chemical messenger nitric oxide (NO), an endothelium derived relaxing factor, is involved in three crucial physiological adaptations of mammalian pregnancy, including vasodilation of the maternal systemic circulation, increased uterine and fetoplacental blood flow, and quiescence of the uterus before parturition [8]. The nitric oxide-soluble guanylyl cyclase-cyclic guanosine monophosphate (NO‐sGc‐cGMP) pathway plays a role in the regulation of fetoplacental circulation in human [9, 10] and oocyte maturation in zebrafish [11]. NO production is stimulated by a variety of mechanical forces such as shear stress [12] and cyclic strain [13] and humoral factors, including Acetylcholine [14], VEGF (Vascular Endothelial Growth Factor) [15], Bradykinin [16, 17], Estrogen [18], S-1P (Sphingosine-1Phosphate) [19, 20], H_2_O_2_ (hydrogen peroxide) [21], and angiotensin [22].

In a previous study, we revealed the adaptation of black rockfish to viviparity based on both the genomic and transcriptomic evidences. We found genes related to placental development, cell adhesion, trophoblast invasion, calcium‐sensing receptors, the NO‐sGc‐cGMP signalling pathway, and blood vessel function were co-expressed in the ovarian wall [4]. We thus hypothesized that the ovarian wall of black rockfish had the function similar to the mammalian uterus. Particularly, we are interested in Bradykinin, which regulates NO production by binding to their cognate receptor. Moreover, we found that bradykinin B2 receptor (*Bdkrb2*) gene family is expanded in black rockfish.

BDKRB2, a component of kallikrein-kinin system (KKS), participates in a wide spectrum of physiological and pathological processes, such as vasodilation, glucose homeostasis, inflammation, and gastric cancer [10, 23–26]. It was reported that the polymorphism of *Bdkrb2* is associated with athletic performance [27] and many diseases, including knee osteoarthritis, diabetic nephropathy, and hypertension [23, 24, 28]. The vasodilation effect of *Bdkrb2* is the result of the synthesis of NO catalyzed by the enzyme nitric oxide synthase (NOS) [29]. *Bdkrb2* was up-regulated during normal pregnancy and detected on human trophoblasts, participating in the establishment and maintenance of placental blood flow through vasodilation, platelet anti-aggregation, cell proliferation, and trophoblast invasion [30, 31]. However, the role of *Bdkrb2* in viviparous teleost is unknown presently Here, we screened and characterized *Bdkrb2* genes in black rockfish and executed phylogenetic and transcriptome analysis to clarify the relationship among the members of *Bdkrb2* gene family. We figured out the primary function of *Bdkrb2* genes in vascular development, which is needed to provide the nutrients to embryos during gestation in viviparous black rockfish. These results suggest that the expansion of *Bdkrb2* gene family is related to adaptation to viviparity in black rockfish. This opens a new window into our understanding of viviparity adaptation in viviparous teleost.

## Results

### Well-developed vascular system in the ovary

**Fig. 1:**
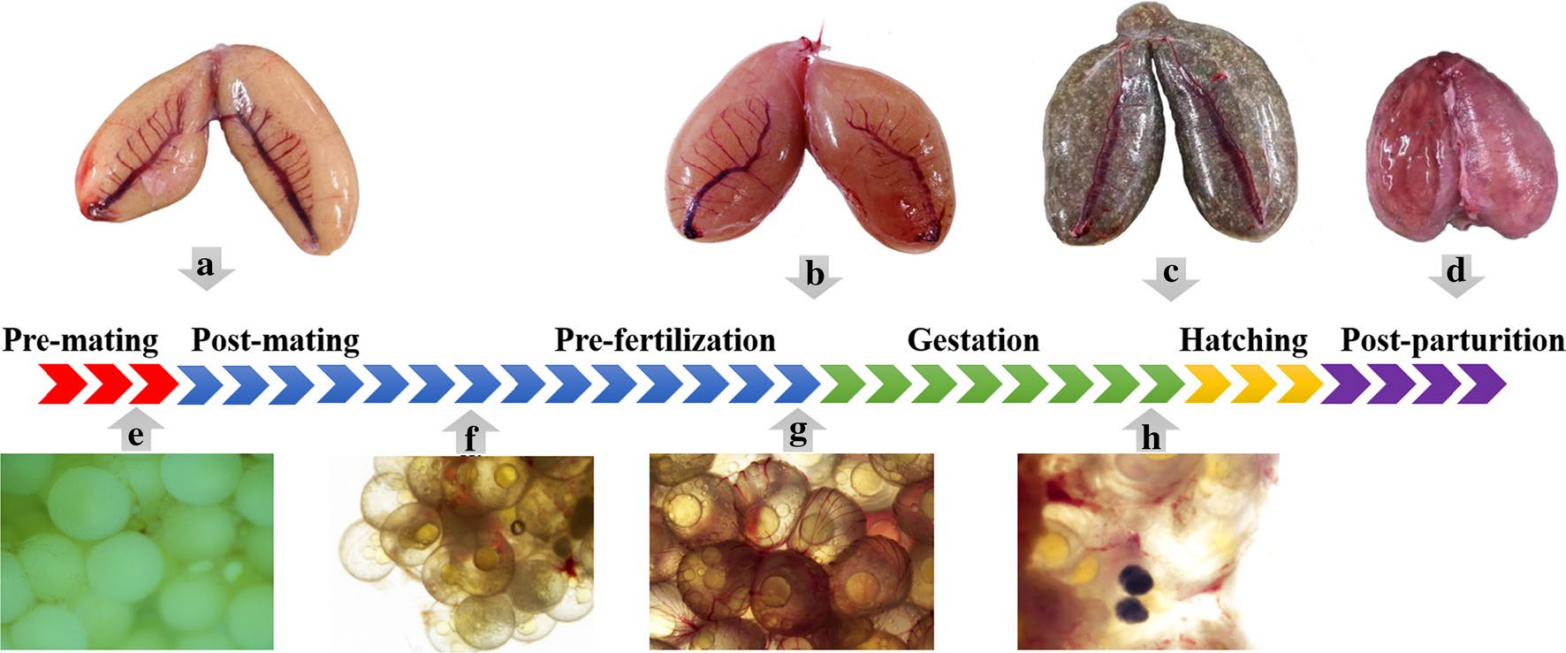
Macroscopic and microcosmic observation of the ovary at different stages of reproductive process. **a** Ovary at the stage of post-mating. Its overall color is yellow, because of fewer blood vessels in the ovary. **b** Ovary after fertilization, at the early stage of gestation. Its overall color is pink or red, because the well-developed blood vessels spread over the surface of egg membrane. **c** Ovary at the final stage of gestation. Its overall color is dark, because of developed pigment. The silvery reflections are the individual eyes of the larvae within the ovary. As time goes by, the ovary will enlarge the volume and the ovarian wall will become very thin until the finish of parturition. **d** Ovary post parturition. Most embryos came out from cloacal orifice via abdomen extrusion. **e** Ovary before mating. Oocytes are opaque. Only a few of blood vessels were observed. **f** Ovary after mating. Oocytes develop further, becoming transparent. **g** Ovary before fertilization. Oocytes are fully developed and ready for fertilization. The highly vascular membranous tissue adjacent to the egg membrane. **h** Ovary during gestation. The eyes of embryo formed

The ovary structure of black rockfish was analyzed for developmental series, from post-mating to post-parturition. The exterior and interior ovarian blood vessels were observed both by eye and microscope after dissection (Fig. 1). Exterior observations showed that a main vessel is located in the middle of ovary wall. Branch vessels and more capillaries extended into the ovary (Fig. 1a–c). Microscopic observations showed that the capillaries spread over the ovary. It is important to note that more interior vessels were observed with the process of final oocyte maturation and gestation (Fig. 1d–h). Highly vascular membranous tissue was found adjacent to the egg membrane, especially in the stages of fertilization and gestation (Fig. 1f–h). Many genes involved in angiogenesis or vasoconstriction had a relatively higher expression in ovarian wall or eggs compared to other genes, which provided the evidence of new vascular formation from molecular level (Additional file 1: Fig. S1 and Additional file 2: Fig. S2). In addition, it is interesting to note that three of five vasoconstriction regulating genes in ovarian wall were *Bdkrb2* genes (Additional file 2: Fig. S2).

### Characterization of *Bdkrb2* genes in black rockfish

**Fig. 2:**
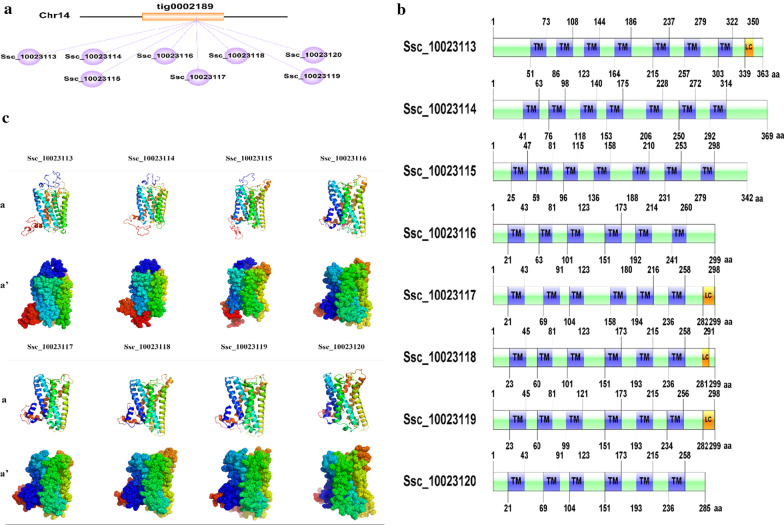
Schematic diagram of gene location and protein structure. **a** Schematic diagram of gene location. **b** Schematic diagram of conserved domains. Domain analysis was performed by SMART online tool base on amino acid sequence. *TM* transmembrane domain, shown in purple, *LC* low complexity domain, shown in orange; other structural region, shown in green. **c** Schematic diagram of 3D structure. Structure prediction analysis was performed via Phyre2 online tool and modified by PyMol software. Models in line a were shown as cartoon style. Models in line a’ were shown as dot style

A total of eight *Bdkrb2* genes were identified in black rockfish genome, including Ssc_10023113, Ssc_10023114, Ssc_10023115, Ssc_10023116, Ssc_10023117, Ssc_10023118, Ssc_10023119 and Ssc_10023120. They were located on chromosome 14, which were arranged in a tandem array, forming a gene cluster spanning about 50 kb (Fig. 2a). Amino acid alignments of *Bdkrb2* genes in black rockfish revealed that Ssc_10023116 was highly homologous to Ssc_10023117 (82%), Ssc_10023118 (84%), Ssc_10023119 (81%) and Ssc_10023120 (78%) (Additional file 3: Table S1). Whereas the similarity among Ssc_10023113, Ssc_10023114 and Ssc_2310023115 was only around 35% (Additional file 4: Fig. S3, Additional file 5: Fig. S4 and Additional file 3: Table S1). The open reading frame (ORF) of Ssc_10023113 was 1092 bp long, encoding 363 amino acids. The ORF of Ssc_10023114 is 1110 bp long, encoding 369 amino acids. The ORF of Ssc_10023115 was 1029 bp long, encoding 342 amino acids. Each one of the BDKRB2 proteins encoded by Ssc_10023113, Ssc_10023114 and Ssc_10023115 contained seven transmembrane (TM) domains, forming a typical G protein (Fig. 2b). The ORF of Ssc_10023120 was 858 bp long, encoding 285 amino acid. The rest four genes, Ssc_10023116, Ssc_10023117, Ssc_10023118, and Ssc_10023119, each contained a 900 bp long ORF and encoded 299 amino acids. Each one of the BDKRB2 proteins encoded by Ssc_10023116, Ssc_10023117, Ssc_10023118, Ssc_10023119 and Ssc_10023120 contained only six TM domains, resulting in protein conformational changes (Fig. 2b, c). Changes were detected by 3D structure prediction. The proteins with seven TM domains had both intracellular and extracellular terminus region, however, the proteins with six TM domains only had intracellular terminus region (Fig. 2c). The conformational changes may cause functional changes.

### Phylogenetic relationship among *Bdkrb2* genes

**Fig. 3:**
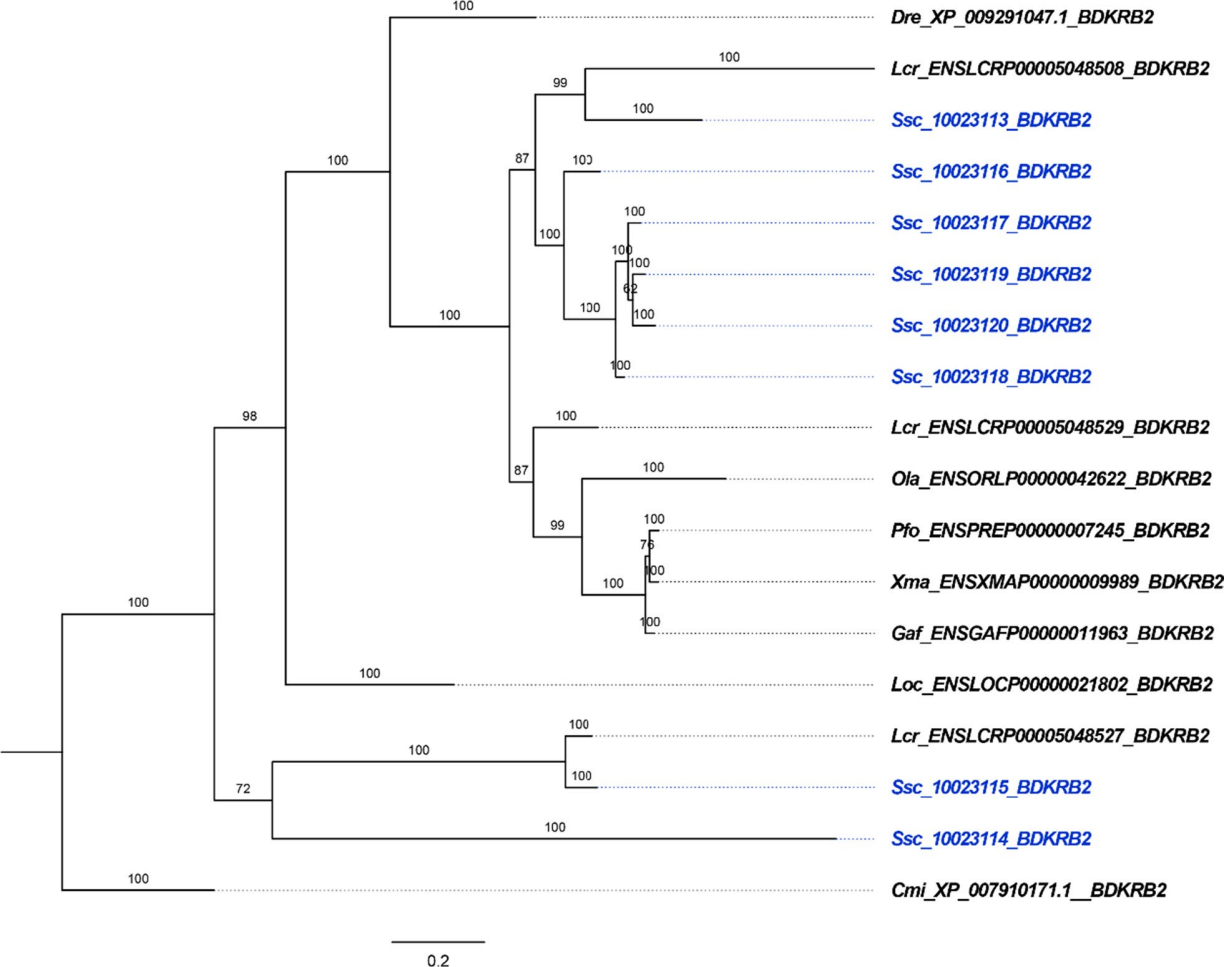
Phylogenetic analysis of *Bdkrb2* paralogs and orthologs in vertebrates. The phylogenetic tree was constructed by MrBayes software. The length of the branch represents genetic distance; posterior probabilities are shown as numbers on the branches; mcmc = 5,000,000; tree was rooted with the sequence of *Callorhinchus milii*. *Cmi, Callorhinchus milii; Dre, Danio Rerio; Lcr, Larimichthys crocea; Loc, Lepisosteus oculatus; Ola, Oryzias latipes; Pfo, Poecilia Formosa; Xma, Xiphophorus maculatus; Ssc, Sebastes schlegelii*

The phylogenetic tree showed that the BDKRB2 orthologs of teleosts were clustered into a clade. It's worth noticing that Ssc_10023113, which had seven TM domains, clustered into a sub-clade with all of the *Bdkrb2* genes only owning six TM domains, including Ssc_10023116, Ssc_10023117, Ssc_10023118, Ssc_10023119 and Ssc_10023120. The remaining two of *Bdkrb2* genes, Ssc_10023114 and Ssc_10023115, which also had seven complete TM domains, were clustered into another sub-clade (Fig. 3).

### Expression and preliminary function analysis of Bdkrb2 genes

**Fig. 4:**
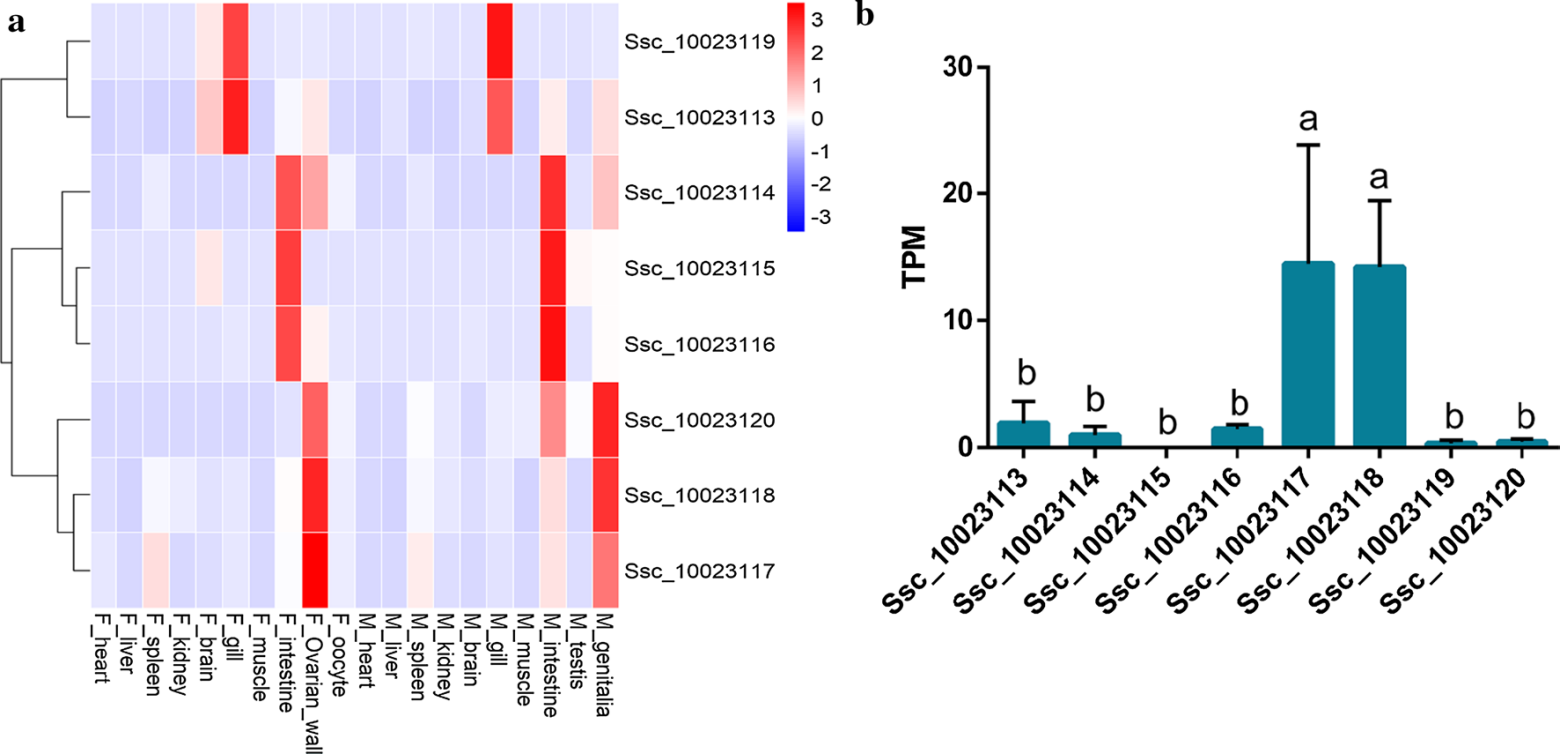
Tissue expression pattern of *Bdkrb2* genes. **a** Heatmap was constructed by comparing different tissues. The x‐axis showed sampled tissues, with the prefix F_ for female and M_ for male samples, and the y‐axis shows genes. The color scale showed standardized TPM values normalized by Z-score method. Gene expression patterns could be roughly divided into gill-type, intestine-type and reproductive organ-type. **b** The expressions of *Bdkrb2* genes in ovarian wall at the stage of post-mating and pre-fertilization. The x‐axis showed genes and the y‐axis shows TPM (Transcripts Per Million). The numeric data were presented as mean ± SD. Statistical significance was tested by one-way ANOVA followed by Tukey’s HSD (harmonic mean sample size = 3, degrees of freedom = 7, subset for alpha = 0.05) using SPSS 20.0 (SPSS, Armonk, NY, USA) and accepted when p < 0.05

To understand the functional differentiation of *Bdkrb2* genes in black rockfish, the expression pattern was analyzed in different tissues. The result showed that eight *Bdkrb2* genes can be roughly classified into three patterns (Fig. 4). Ssc_10023113 and Ssc_10023119 were highly expressed in gills. Ssc_10023114, Ssc_10023115 and Ssc_10023116 were highly expressed in intestines, regardless of sex. Ssc_10023117, Ssc_10023118 and Ssc_10023120 were highly expressed in the ovarian wall in females and genitalia in males (Fig. 4a). Among the eight genes, Ssc_10023113 had the widest tissue expression, including the brain, gill, intestine, ovarian wall and genitalia. Expressions of *Bdkrb2* genes in the ovarian wall were analyzed in detail. Ssc_10023117 and Ssc_10023118 had a significant higher expression in the tissue of ovarian wall at the stage of post_mating and pre_fertilization (Fig. 4b).

**Fig. 5:**
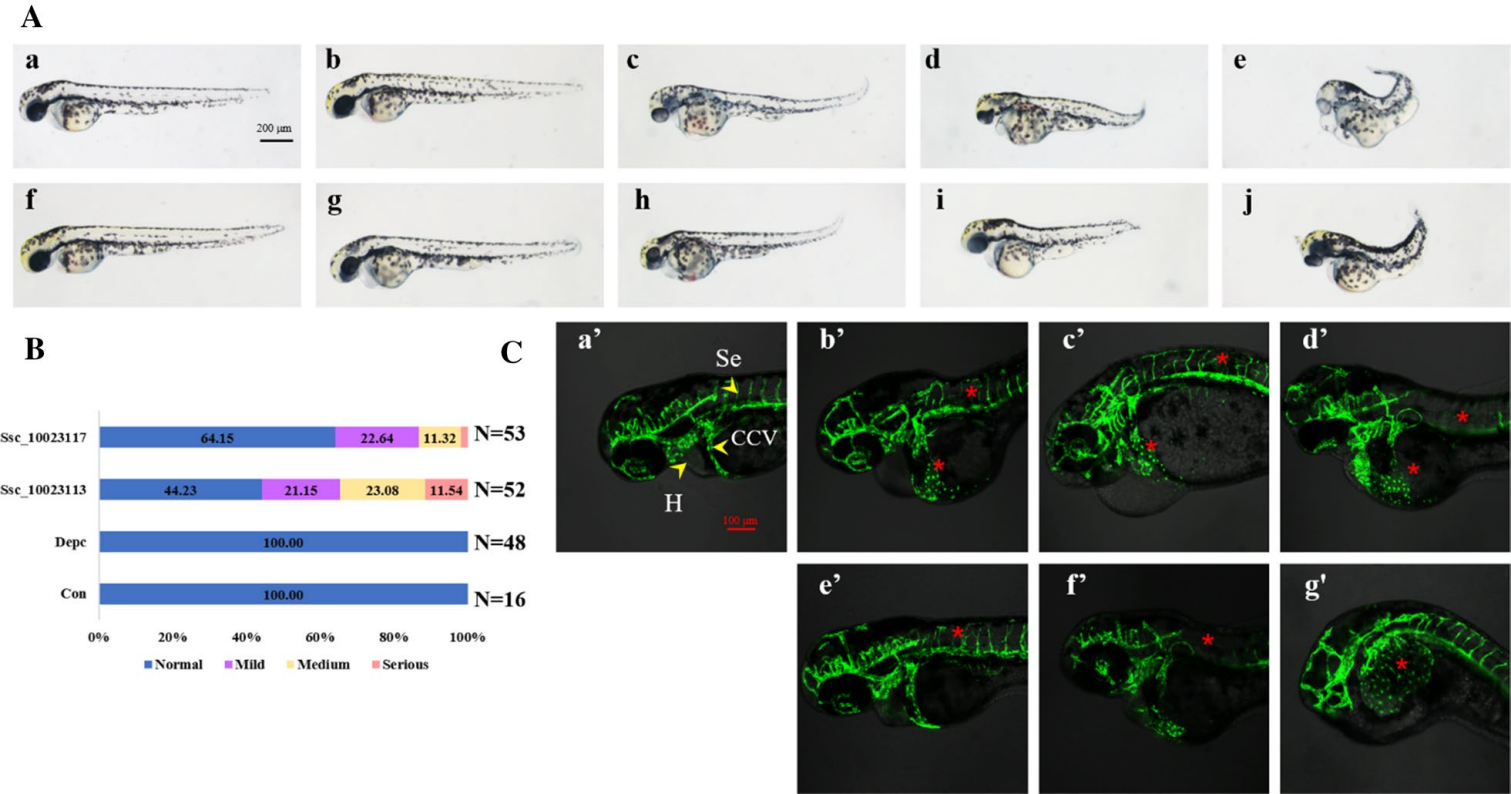
Functional study of *bdkrb2* genes in zebrafish. Overexpression of Ssc_10023113 and Ssc_10023117 affects the phenotype and vascular development of embryos. An amount of 1.5 ng/embryo of mRNA was injected into Transgenic stain (*Fli1a*: EGFP) embryos at 1–4 cell stages. All embryos are shown with anterior to the left. **a** Phenotype of embryos with overexpression of mRNA at 48 h post-fertilization (hpf). Scale bar = 200 μm. **a** control group, embryos without injection. **f** control group, embryos with injection of equal volume of sterilized DEPC water. **B**–**e**, experimental group, embryos with injection of Ssc_10023113 mRNA. **b** normal phenotype; **c** mild phenotype; **d** medium phenotype; **d** serious phenotype. **g**–**j**, experimental group, embryos with injection of Ssc_10023117 mRNA. **g** normal phenotype; **h** mild phenotype; **i** medium phenotype; **j** serious phenotype. **b** Deformation percentages of embryos in each category as shown in (**a**). Phenotypes were divided into four categories: normal, shown in blue; mild, shown in purple; medium, shown in yellow; serious, shown in pink. N is the number of total samples analyzed in each group. **c** Vascular abnormalities of embryos with overexpression of mRNA at about 55 hpf. Scale bar = 100 μm. **a**’, control group, embryos with injection of equal volume of sterilized DEPC water. **b**’-**d**’, experimental group, embryos with injection of Ssc_10023113 mRNA. **e**’–**g**’, experimental group, embryos with injection of Ssc_10023117 mRNA. Yellow arrowheads indicate different types of vessels, whereas the red asterisks point out the abnormalities in the experimental group. *H* heart, *CCV* common cardinal veins, *Se* intersegmental vessel

To explore the function of *Bdkrb2* genes, Ssc_10023113 and Ssc_10023117 was chosen as the representatives of genes, which encoded BDKRB2 protein with seven and six transmembrane domains, respectively. Capped mRNA of two genes were synthesized in vitro. Transgenic zebrafish strain (*Fli1a*: EGFP) was used for functional study due to technical limitations in the black rockfish. Both overexpression of Ssc_10023113 and Ssc_10023117 resulted in different degrees of malformation in 24 h post-fertilization (hpf) embryos, including rostral-caudal axis, edema of the pericardial cavity (Fig. 5a). The rates of deformation were 55.77% and 35.65% for Ssc_10023113 and Ssc_10023117 genes, respectively, showing no statistical significance by chi-square test (P = 0.368). Confocal microscopy observation showed that overexpression of Ssc_10023113 and Ssc_10023117 both resulted in abnormal vascular development, including increased diameter of the common cardinal veins (CCVs), anomalous and undifferentiated intersegmental vessel (Se). It indicates that Ssc_10023113 and Ssc_10023117 had similar function in regulation of embryo development and vascular formation.

### Bdkrb2 genes play a role in the ovarian wall in the reproductive cycle

**Fig. 6:**
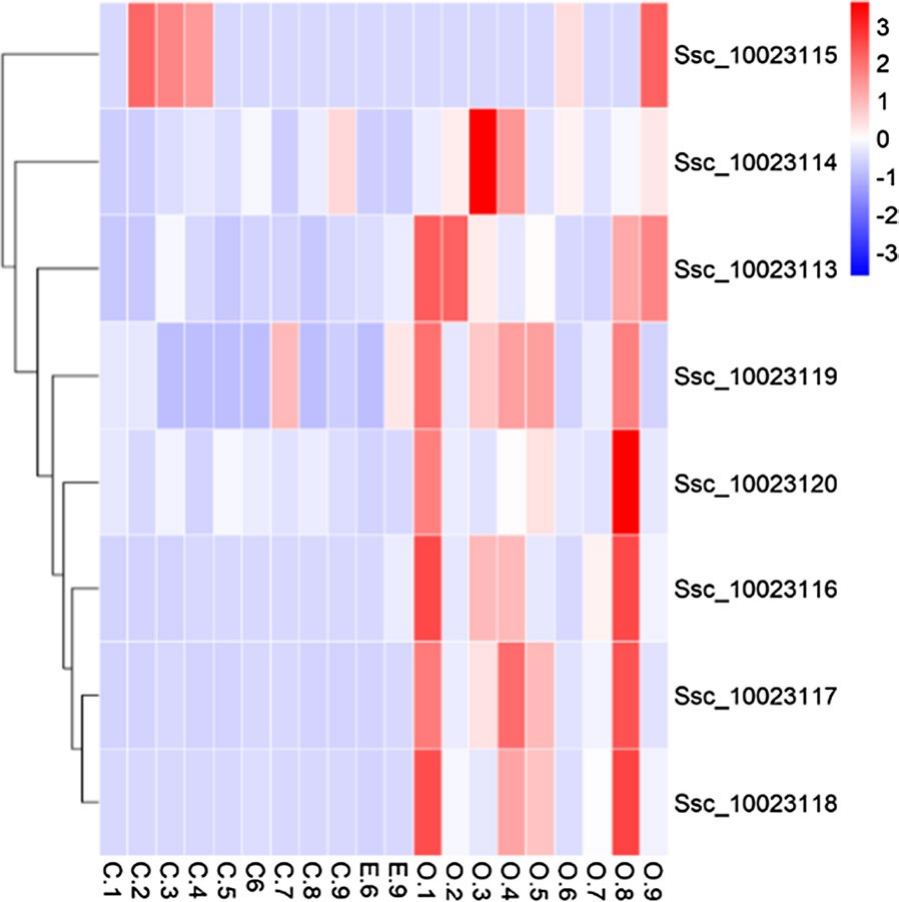
Expression pattern of *Bdkrb2* genes in the ovary in the reproductive cycle. **a** Heatmap was constructed by comparing three kinds of samples at different stage of the reproductive cycle. The x‐axis shows sampled tissues with the prefix C. for connective tissue rich in blood vessels covering the egg membrane and E. for embryos and O. for ovarian wall. Arabic numerals represent different stages. 1, pre-fertilization; 2, 1-cell; 3, 8-cells; 4, 16-cells; 5, gastrula stage; 6, 8-somites stage; 7, tailbud stage; 8, pre-hatching; 9, hatching. The color scale shows standardized TPM values normalized by Z-score method

To investigate the function of *Bdkrb2* genes in the ovary in the reproductive cycle, samples of connective tissue rich in blood vessels covering the egg membrane, embryos and ovarian wall at different stages were collected for RNA-seq. The expression analysis provided more detailed information about the different regulatory pattern of angiogenesis and vasoconstriction in the three kinds of samples (Additional file 6: Fig. S5 and Additional file 7: Fig. S6). Angiogenesis and vasoconstriction related genes have continuous expression at pre-fertilization and gestation. All *Bdkrb2* genes are expressed in the ovarian wall, however, their expression periods were not similar (Fig. 6). Jonckheere–Terpstra Test was performed to demonstrate that mean expression of selected gene differed among the developmental stages. The result showed that the expression level of Ssc_10023113 (P = 0.006), Ssc_10023114 (P = 0.015), Ssc_10023116 (P = 0.000), Ssc_10023117 (P = 0.001), Ssc_10023118 (P = 0.001) or Ssc_10023119 (P = 0.038) was significantly different among the 20 stages sampled. Ssc_10023113, Ssc_10023116, Ssc_10023117, Ssc_10023118, Ssc_10023119 and Ssc_10023120 had an expression at the stage of pre-fertilization and pre-hatching, suggesting that these genes play a role in preparing for fertilization and hatching. Furthermore, Ssc_10023113, Ssc_10023114 and Ssc_10023115 also highly expressed at the stages at the stage of hatching, suggesting that these genes play a role in hatching.

## Discussion

In this study, we identified eight *Bdkrb2* genes from the black rockfish genome, which showed the expanded gene number of this family. The BDKRB2 protein is encoded by a single-copy gene in human, which contains 3 exons separated by 2 introns. The first and second exons are noncoding, while the third exon contains the full-length coding region [32]. Black rockfish has the highest number of *Bdkrb2* genes of the species we investigated. BDKRB2 is a G protein receptor, which functions by binding the ligand bradykinin via N-terminus and stimulating PI3K/Akt via intracellular domains to activate Calmodulin, leading to efficient nitric oxide (NO) synthesis [16, 17]. The conformational changes, lacing one TM domain, can cause functional changes. The interaction with ligand may be affected in the proteins with six TM domains, because both N-terminus and C-terminus of these proteins are intracellular. Here, we provide a perspective that BDKRB2 with six TM domains (encoded by Ssc_10023116, Ssc_10023117, Ssc_10023118, Ssc_10023119 or Ssc_10023120) could amplify intracellular signals when co-expressed with BDKRB2 with seven TM domains (encoded by Ssc_10023113, Ssc_10023114 or Ssc_10023115). More studies on protein structure, location and function need to be further explored.

It is surprising to see that the three genes, that contain the complete seven TM were not clustered to the same sub-clade. The sub-clade grouped by Ssc_10023113 and the other five genes with only six TM domains demonstrated that these six genes were more conserved. Based on this, we hypothesized that the five “truncated” genes were duplicated from Ssc_10023113 and lost one TM domain during evolution.

The tissue expression pattern showed that eight *Bdkrb2* genes can be roughly divided into three patterns, including gill-biased, intestine-biased and ovarian wall- or genitalia-biased types, suggesting the possibility of functional differentiation of these genes.

Interestingly, *Bdkrb2* genes had biased expression in ovarian wall, but not in oocytes of pre-fertilization adults. The tissue RNA-sequencing data of human (data from NCBI database, accession ID: PRJNA280600), house mouse (data from NCBI database, accession ID: PRJNA66167) and Norway rat (data from NCBI database, accession ID: PRJNA238328) showed that *Bdkrb2* gene had a relatively high expression in uterus and placenta, but a relatively low expression in ovary and testis in mammals. Both human and rat BDKRB2 proteins show a high expression on the membrane of the epithelial cells of endometrial glands and luminal [33]. A greater expression of BDKRB2 protein was observed in the early pregnancy samples and the location of signals in the uterus coincide with other vasoactive effectors such as NO, prostacyclin, growth factors and renin [34, 35]. The expression of *Bdkrb2* was also detected in human endometrial and prostate cancers [36]. These studies suggested that *Bdkrb2* is related to the physiological and pathological functions of the uterus. Uterus is the organ where fetus develop. Placenta is developmentally crucial for reproductive success in placental mammals [37]. Unlike the placental mammals, black rockfish did not evolve a uterus, whose embryos develop in the ovary. The expression in of *Bdkrb2* genes in ovarian wall but not in oocytes suggests that ovarian wall gain the function similar to mammalian uterus to meet the need of gestation.

*Bdkrb2* genes also had biased expression in genitalia. Previous studies have shown the expression of kallikrein-kinin system (KKS) components in the male reproductive tract and the function related to smooth-muscle contraction, prostaglandin production, the motility and vitality of ejaculated spermatozoa and anion secretion [38–41]. *Bdkrb2*, an important component of KKS, showed segmental expression in rat efferent ducts and epididymis and played a role in glycerol and potentially water transport via AQP9, which was essential for concentration of spermatozoa and the establishment of luminal hypertonicity [42]. These data point to a regulatory role for *Bdkrb2* in sperm maturation and storage. One significant character of black rockfish is long-term storage of sperm in the ovary. However, little has been known about the mechanism. Whether *Bdkrb2* participates in the sperm preparation need further investigation.

Preliminary functional experiment in zebrafish showed that Ssc_10023113 and Ssc_10023117 had a similar function in embryo development, though the protein encoded by Ssc_10023113 contains seven TM domains and that encoded by Ssc_10023117 only contains six TM domains. Moreover, over-expression of Ssc_10023113 and Ssc_10023117 mRNA resulted in similar vascular abnormalities. The development of intersegmental vessels (Se) and/ or common cardinal veins (CCVs) was affected. *Bdkrb2* was one of genes related to cerebrovascular dysfunction with the development of Alzheimer’s disease-like pathology in OXYS rats. Its expression was found to be reduced at 20-day-old and 18-month-old, and was increased at 5-month-old in OXYS rats [43]. However, the underlying mechanisms need further research.

We previously reported that the NO‐sGc‐cGMP signaling pathway was identified in WGCNA co-expression module of ovarian wall, and proposed that the rockfish ovarian wall has a similar function to the uterus of mammals [4]. The chemical messenger NO is an endothelium derived relaxing factor, which participates in the control of vasorelaxation in fetoplacental vessels [44]. *Bdkrb2* can participate in regulating vasodilation, vasopermeability, matrix degradation cell proliferation, and myometrial contractility by controlling NO production via binding with bradykinin in the key sites for embryo attachment, implantation, placentation, maintenance of placental blood flow, and parturition [35]. The data of RNA-seq showed that the eight *Bdkrb2* genes have time-dependent expression in the ovarian wall during the reproduction cycle. Furthermore, Ssc_10023113, Ssc_10023116, Ssc_10023117, Ssc_10023118, Ssc_10023119 and Ssc_10023120 were relatively high expressed in the ovarian wall at the stage of pre-fertilization and pre-hatching, suggesting that these genes play a regulatory role in the preparation for fertilization and hatching.

## Conclusion

In this work, we characterized eight *Bdrkb2* genes in the black rockfish, which have a regulatory role in the preparation for fertilization and hatching. Our study suggests that the expansion of *Bdkrb2* gene family is an adaptation of black rockfish to viviparity.

## Methods

### Fish and samples

Black rockfish (*Sebastes schlegelii*) were obtained from Zhucha Island (Qingdao, Shandong, China). Samples used for tissues RNA‐seq were collected in December 2017 as reported in our previous study [4]. Six healthy 3-year-old fish (three males and three females) at the stage of post_mating and pre_fertilization was randomly selected for sampling of heart, liver, spleen, kidney, brain, intestine, gill, muscle, ovarian wall, male genitalia, oocytes and gonad (testis or ovary). 12 pregnant 3-year-old female fish were captured from the deep-sea cage and transported to the lab at Ocean University of China in late March. The fish were raised in the lab for three to 21 days and then dissected for sampling the embryos, ovarian wall and connective tissue rich in blood vessels covering the egg membrane. The developmental stages of embryos were determined under microscope. Three embryos at somite and three juveniles at pre-hatching stage were also collected. A total of 30 transcriptomes were generated. The sampling size at different developmental stages and samples used for RNA-seq was listed in the table. S2 (Additional file 8).

### Transcriptome data

The RNA-seq data of different tissues was obtained from our previous study [4], which was deposited in CNSA (CNGB Nucleotide sequence archive) with the accession ID CNP0000222. The ovarian RNA-seq data was generated in this study, the data of interested genes was shown in Additional file 9. Total RNA of different parts of the ovary and embryos at different developmental stages was extracted with Trizol Reagent (Invitrogen, Carlsbad, USA) according to the manufacture’s protocol. RNA quality was assessed using an Agilent 2100 Bioanalyzer. RNA‐seq libraries were constructed by MGIEasy Universal Library Conversion Kit (APP-A) V1.0 and then 100-bp paired-end reads were generated on BGI-seq 500 platform. Averaged 175.26 Mb raw reads per sample were obtained in ovarian tissues RNA-seq. The resulting raw data were filtered to remove adapter sequences, empty reads, and low-quality reads by SOAPnuke [45]. Averaged 163.59 Mb clean reads per sample were obtained, respectively (Additional file 8: Table. S2). The transcriptomic data were mapped to the rockfish genome and the expression level of genes was calculated using Salmon [46] with default parameters. The mean TPM (Transcripts Per Million) values of genes of interest were subsequently visualized via the Heatmap package [47]. To demonstrate that mean expression of selected genes differs among the developmental stages, Jonckheere-Terpstra test, which test for ordered differences among samples, was executed by SPSS 20 with default parameters.

### Sequence alignment and protein structure analysis

Sequence alignments and comparisons of homology between eight *Bdkrb2* ORF sequences or amino acid sequences were performed using the MUSCLE algorithm in MEGAX with default parameters [48]. The protein domains were predicted by SMART (available online: http://smart.embl-heidelberg.de) [49] and the schematic diagram was constructed by the online tool IBS (available online: http://ibs.biocuckoo.org/online.php) [50]. 3D Structure prediction analysis was performed via Phyre2 online tool [51] and modified by PyMol software (https://pymol.org/2/).

### Phylogenetic analysis

To elucidate the evolutionary relationship of the eight *Bdkrb2* genes in black rockfish, a phylogenetic tree was constructed based on amino acid sequences of the *Bdkrb2* or *Bdkrb2-like* genes from 9 species of fish. Cartilaginous fish (*Callorhinchus milii*), bony fishes (*Danio Reri, Larimichthys crocea, Lepisosteus oculatus, Oryzias latipes, Poecilia Formosa, Xiphophorus maculatus, Sebastes schlegelii*) were included in the phylogenetic analysis. The evolutionary history was inferred by using the Mrbayes software [52]. All the gaps produced by multiple sequence alignment were not deleted for construction of MrBayes tree. Bayesian analysis was run for 500,000 generations with a burn-in of 25% and default Metropolis coupling parameters (sample frequency = 500 generations, standard deviation of split partitions < 0.01). This analysis involved 18 amino acid sequences, which were retrieved from NCBI (available online: http://www.ncbi.nlm.gov) and Ensemble (available online: www.ensembl.org). *Callorhinchus milii* (BDKRB2, XP_007910171.1), *Danio rerio* (Bdkrb2_like, XP_009291047.1), *Larimichthys crocea* (BDKRB2, ENSLCRP00005048508, ENSLCRP00005048527, ENSLCRP00005048529), *Lepisosteus oculatus* (BDKRB2, ENSLOCP00000021802), *Oryzias latipes* (BDKRB2, ENSORLP00000042622), *Poecilia formosa* (BDKRB2, ENSPREP00000007245), *Gambusia affinis* (BDKRB2, ENSGAFP00000011963), *Xiphophorus maculatus* (BDKRB2, ENSXMAP00000009989) and *Sebastes schlegelii* (Ssc_100231113, Ssc_100231114, Ssc_100231115, Ssc_100231116, Ssc_100231117, Ssc_100231118, Ssc_100231119 and Ssc_100231120) from local data (Additional file 5: Fig. S4).

### mRNA synthesis and microinjection

Capped mRNAs of two *Bdkrb2* genes (Ssc_10023113 and Ssc_10023117) were synthesized with mMESSAGE mMACHINE@T7 (Ambion, Foster City, CA, USA). The primers were Ssc_10023113_mRNA_Fw/ Rv and Ssc_10023117_mRNA_Fw/Rv (Additional file 9: Table S3). Microinjection was performed on arvard Apparatus PLI-100 (NatureGene, NV, USA) machine in one- to four-cell-stage embryos of transgenic strain (*Fli1a*: EGFP) with 1.5 ng *Bdkrb2* mRNA of black rockfish for each embryo. Embryos without injection and with injection of sterilized DEPC water were the control groups.

## Supplementary Information

**Additional file 1: Fig. S1.** Tissue expression pattern of genes involved in angiogenesis. Heatmap was constructed by comparing 20 tissues. The x‐axis shows sampled tissues, with the prefix F_ for female and M_ for male samples, and the y‐axis shows genes. The color scale shows standardized TPM values normalized by Z-score method.

**Additional file 2: Fig. S2.** Tissue expression pattern of genes involved in regulation of vasoconstriction. Heatmap was constructed by comparing 20 tissues. The x‐axis shows sampled tissues, with the prefix F_ for female and M_ for male samples, and the y‐axis shows genes. The color scale shows standardized TPM values normalized by Z-score method. Five genes (Ssc_10023120, Ssc_1004710, Ssc_10011522, Ssc_10023117 and Ssc_10023118) have a bias expression in ovarian wall and genitalia, three of which are *Bdkrb2* genes (Ssc_10023117, Ssc_10023118 and Ssc_10023120).

**Additional file 3: Table S1**. Sequence similarity of *Bdkrb2* genes in black rockfish.

**Additional file 4: Fig. S3.** Similarity comparison of nucleotide sequences of the *Bdkrb2* ORF in black rock fish. Ssc_13, Ssc_10023113; Ssc_14, Ssc_10023114; Ssc_15, Ssc_10023115; Ssc_16, Ssc_10023116; Ssc_17, Ssc_10023117; Ssc_18, Ssc_10023118; Ssc_19, Ssc_10023119; Ssc_20, Ssc_10023120. The results are shaded to four levels. Dark shadows indicate very high conservative amino acid residues.

**Additional file 5: Fig. S4.** Similarity comparison of amino acid sequences of the *Bdkrb2* ORF in black rock fish. Ssc_13, Ssc_10023113; Ssc_14, Ssc_10023114; Ssc_15, Ssc_10023115; Ssc_16, Ssc_10023116; Ssc_17, Ssc_10023117; Ssc_18, Ssc_10023118; Ssc_19, Ssc_10023119; Ssc_20, Ssc_10023120. The results are shaded to four levels. Dark shadows indicate very high conservative amino acid residues.

**Additional file 6: Fig. S5.** Expression pattern of genes involved in regulation of angiogenesis in the ovary in the reproductive cycle. A) Heatmap was constructed by comparing three kinds of samples at different stage of the reproductive cycle. The x‐axis shows sampled tissues with the prefix C. for connective tissue rich in blood vessels covering the egg membrane and E. for embryos and O. for ovarian wall. Arabic numerals represent different stages. 1, pre-fertilization; 2, 1-cell; 3, 8-cells; 4, 16-cells; 5, gastrula stage; 6, 8-somites stage; 7, tailbud stage; 8, pre-hatching; 9, hatching.

**Additional file 7: Fig. S6.** Expression pattern of genes involved in regulation of vasoconstriction in the ovary in the reproductive cycle. A) Heatmap was constructed by comparing three kinds of samples at different stage of the reproductive cycle. The x‐axis shows sampled tissues with the prefix C. for connective tissue rich in blood vessels covering the egg membrane and E. for embryos and O. for ovarian wall. Arabic numerals represent different stages. 1, pre-fertilization; 2, 1-cell; 3, 8-cells; 4, 16-cells; 5, gastrula stage; 6, 8-somites stage; 7, tailbud stage; 8, pre-hatching; 9, hatching.

**Additional file 8: Table S2. ** Sample and data statistics of ovarian RNA-seq.

**Additional file 9. Table S3.** Expression levels (TPM; Transcripts Per Million) of metioned genes in different tissues of black rockfish. The prefix C indicated for connective tissue rich in blood vessels covering the egg membrane and E for embryos and O for ovarian wall. Arabic numerals represent different stages. 1, prefertilization; 2, 1-cell; 3, 8-cells; 4, 16-cells; 5, gastrula stage; 6, 8-somites stage; 7, tailbud stage; 8, pre-hatching; 9, hatching.

## Data Availability

The datasets of whole genome sequencing of Sebastes schlegelii and tissues RNA-seq were deposited in CNSA (CNGB Nucleotide sequence archive) with the accession ID CNP0000222. The ovarian RNA-seq datasets, which was used for other unpublished study, the data analyzed during this study are included in additional file or are available from the corresponding author on reasonable request.

## Abbreviations

Bdkrb2: Bradykinin receptor B2
NO: Nitric oxide
sGC: Soluble guanylyl cyclase
cGMP: Cyclic guanosine monophosphate
VEGF: Vascular endothelial growth factor
S-1P: Sphingosine-1phosphate
H_2_O_2_: Hydrogen peroxide
KKS: Kallikrein-kinin system
NOS: Nitric oxide synthase
eNOS: Endothelial nitric oxide synthase
ORF: Open reading frame
TM: Transmembrane
CCVs: Common cardinal veins
Se: Intersegmental vessel
TPM: Transcripts per million
hpf: Hour post-fertilization
