# Supplementary material for: The Bdkrb2 gene family provides a novel view of viviparity adaptation in Sebastes schlegelii
Source: BMC Ecol Evol. 2021 Mar 17;21:44. doi: 10.1186/s12862-021-01774-0 (PMC7968187; doi:10.1186/s12862-021-01774-0)
Supplement: Supplementary file 1 — Additional file 1: Fig. S1. Tissue expression pattern of genes involved in angiogenesis. Heatmap was constructed by comparing 20 tissues. The x‐axis shows sampled tissues, with the prefix F_ for female and M_ for male samples, and the y‐axis shows genes. The color scale shows standardized TPM values normalized by Z-score method. [file 12862_2021_1774_MOESM1_ESM.docx]

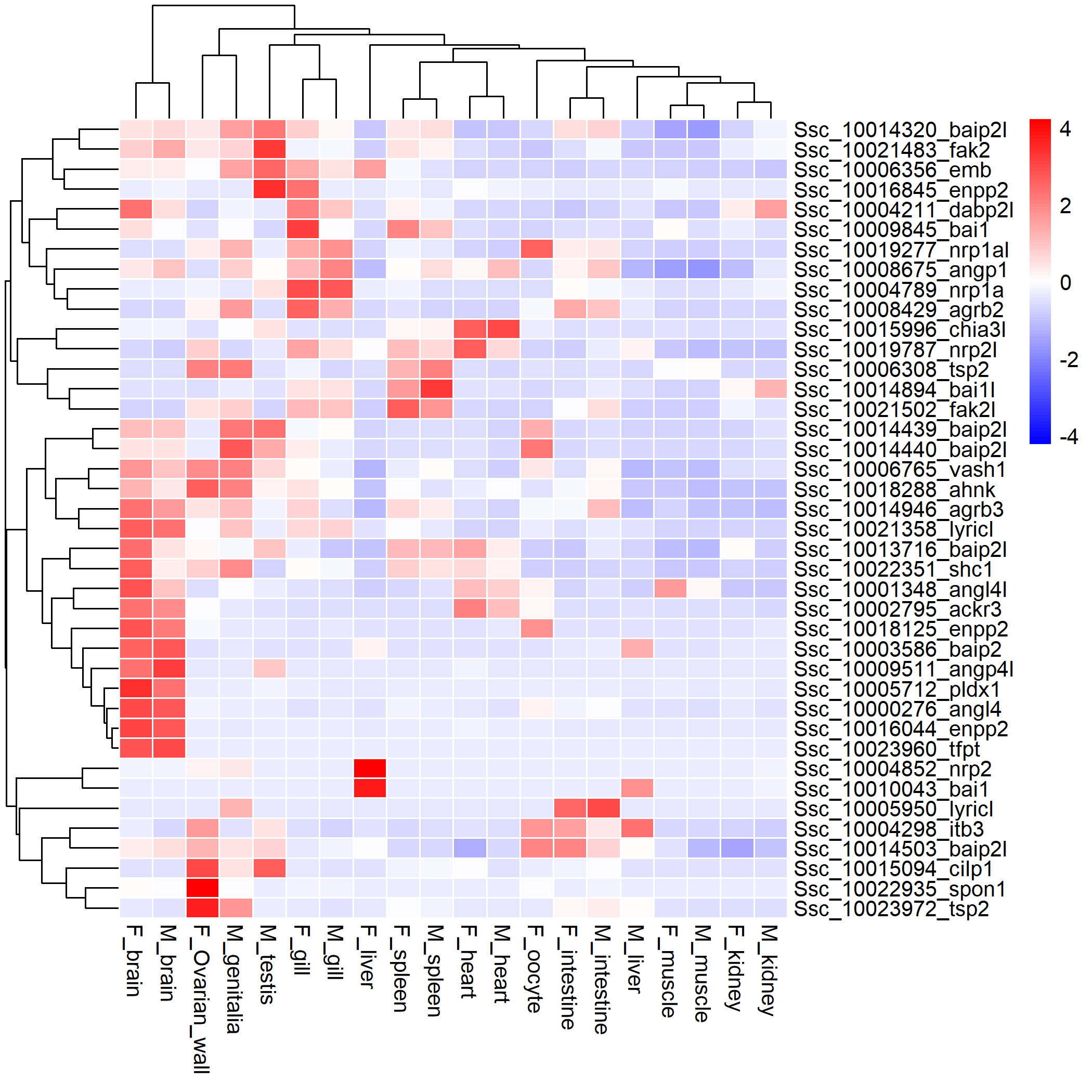


**Fig.S1** Tissue expression pattern of genes involved in angiogenesis. Heatmap was constructed by comparing 20 tissues. The x‐axis shows sampled tissues, with the prefix F_ for female and M_ for male samples, and the y‐axis shows genes. The color scale shows standardized TPM values normalized by Z-score method.
