# Supplementary material for: The Bdkrb2 gene family provides a novel view of viviparity adaptation in Sebastes schlegelii
Source: BMC Ecol Evol. 2021 Mar 17;21:44. doi: 10.1186/s12862-021-01774-0 (PMC7968187; doi:10.1186/s12862-021-01774-0)
Supplement: Supplementary file 2 — Additional file 2: Fig. S2. Tissue expression pattern of genes involved in regulation of vasoconstriction. Heatmap was constructed by comparing 20 tissues. The x‐axis shows sampled tissues, with the prefix F_ for female and M_ for male samples, and the y‐axis shows genes. The color scale shows standardized TPM values normalized by Z-score method. Five genes (Ssc_10023120, Ssc_1004710, Ssc_10011522, Ssc_10023117 and Ssc_10023118) have a bias expression in ovarian wall and genitalia, three of which are Bdkrb2 genes (Ssc_10023117, Ssc_10023118 and Ssc_10023120). [file 12862_2021_1774_MOESM2_ESM.docx]

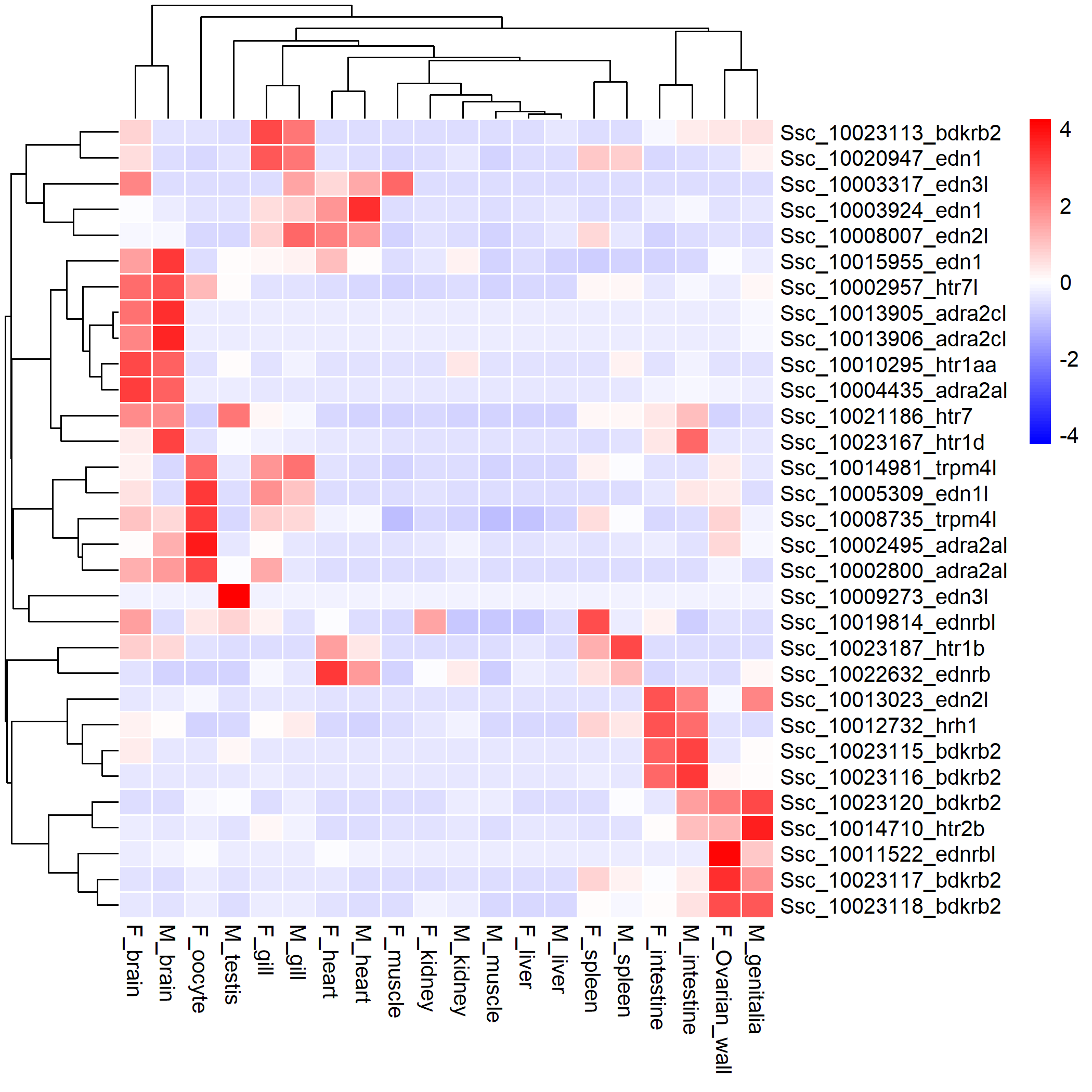


**Fig. S2** Tissue expression pattern of genes involved in regulation of vasoconstriction. Heatmap was constructed by comparing 20 tissues. The x‐axis shows sampled tissues, with the prefix F_ for female and M_ for male samples, and the y‐axis shows genes. The color scale shows standardized TPM values normalized by Z-score method. Five genes (Ssc_10023120, Ssc_1004710, Ssc_10011522, Ssc_10023117 and Ssc_10023118) have a bias expression in ovarian wall and genitalia, three of which are *Bdkrb2* genes (Ssc_10023117, Ssc_10023118 and Ssc_10023120).
