# Supplementary material for: The Bdkrb2 gene family provides a novel view of viviparity adaptation in Sebastes schlegelii
Source: BMC Ecol Evol. 2021 Mar 17;21:44. doi: 10.1186/s12862-021-01774-0 (PMC7968187; doi:10.1186/s12862-021-01774-0)
Supplement: Supplementary file 3 — Additional file 3: Table S1. Sequence similarity of Bdkrb2 genes in black rockfish. [file 12862_2021_1774_MOESM3_ESM.docx]

**Table. S1 Sequence similarity of *Bdkrb2* genes in black rockfish**

|  | Ssc_10023113 | Ssc_10023114 | Ssc_10023115 | Ssc_10023116 | Ssc_10023117 | Ssc_10023118 | Ssc_10023119 | Ssc_10023120 |
| --- | --- | --- | --- | --- | --- | --- | --- | --- |
| Ssc_10023113 | 100% | 34% | 36% | 57% | 59% | 58% | 58% | 57% |
| Ssc_10023114 |  |  | 33% | 36% | 33% | 34% | 33% | 32% |
| Ssc_10023115 |  |  |  | 39% | 36% | 35% | 35% | 35% |
| Ssc_10023116 |  |  |  |  | 82% | 84% | 81% | 78% |
| Ssc_10023117 |  |  |  |  |  | 94% | 95% | 89% |
| Ssc_10023118 |  |  |  |  |  |  | 93% | 87% |
| Ssc_10023119 |  |  |  |  |  |  |  | 89% |
| Ssc_10023120 |  |  |  |  |  |  |  | 100% |
