# Supplementary material for: The Bdkrb2 gene family provides a novel view of viviparity adaptation in Sebastes schlegelii
Source: BMC Ecol Evol. 2021 Mar 17;21:44. doi: 10.1186/s12862-021-01774-0 (PMC7968187; doi:10.1186/s12862-021-01774-0)
Supplement: Supplementary file 4 — Additional file 4: Fig. S3. Similarity comparison of nucleotide sequences of the Bdkrb2 ORF in black rock fish. Ssc_13, Ssc_10023113; Ssc_14, Ssc_10023114; Ssc_15, Ssc_10023115; Ssc_16, Ssc_10023116; Ssc_17, Ssc_10023117; Ssc_18, Ssc_10023118; Ssc_19, Ssc_10023119; Ssc_20, Ssc_10023120. The results are shaded to four levels. Dark shadows indicate very high conservative amino acid residues. [file 12862_2021_1774_MOESM4_ESM.docx]

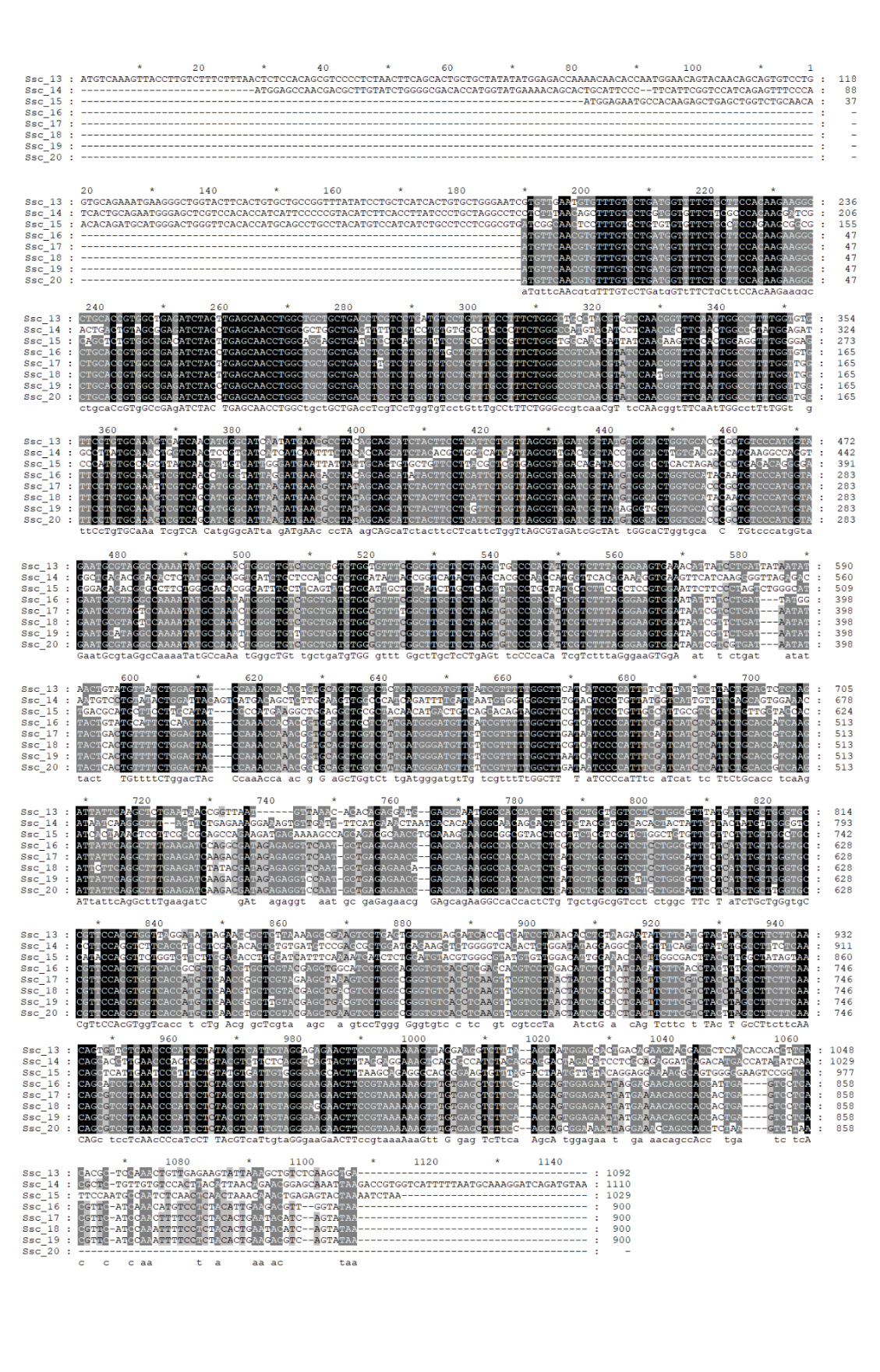


**Fig. S3** Similarity comparison of nucleotide sequences of the *Bdkrb2* ORF in black rock fish. Ssc_13, Ssc_10023113; Ssc_14, Ssc_10023114; Ssc_15, Ssc_10023115; Ssc_16, Ssc_10023116; Ssc_17, Ssc_10023117; Ssc_18, Ssc_10023118; Ssc_19, Ssc_10023119; Ssc_20, Ssc_10023120. The results are shaded to four levels. Dark shadows indicate very high conservative amino acid residues.
