# Supplementary material for: The Bdkrb2 gene family provides a novel view of viviparity adaptation in Sebastes schlegelii
Source: BMC Ecol Evol. 2021 Mar 17;21:44. doi: 10.1186/s12862-021-01774-0 (PMC7968187; doi:10.1186/s12862-021-01774-0)
Supplement: Supplementary file 6 — Additional file 6: Fig. S5. Expression pattern of genes involved in regulation of angiogenesis in the ovary in the reproductive cycle. A) Heatmap was constructed by comparing three kinds of samples at different stage of the reproductive cycle. The x‐axis shows sampled tissues with the prefix C. for connective tissue rich in blood vessels covering the egg membrane and E. for embryos and O. for ovarian wall. Arabic numerals represent different stages. 1, pre-fertilization; 2, 1-cell; 3, 8-cells; 4, 16-cells; 5, gastrula stage; 6, 8-somites stage; 7, tailbud stage; 8, pre-hatching; 9, hatching. [file 12862_2021_1774_MOESM6_ESM.docx]

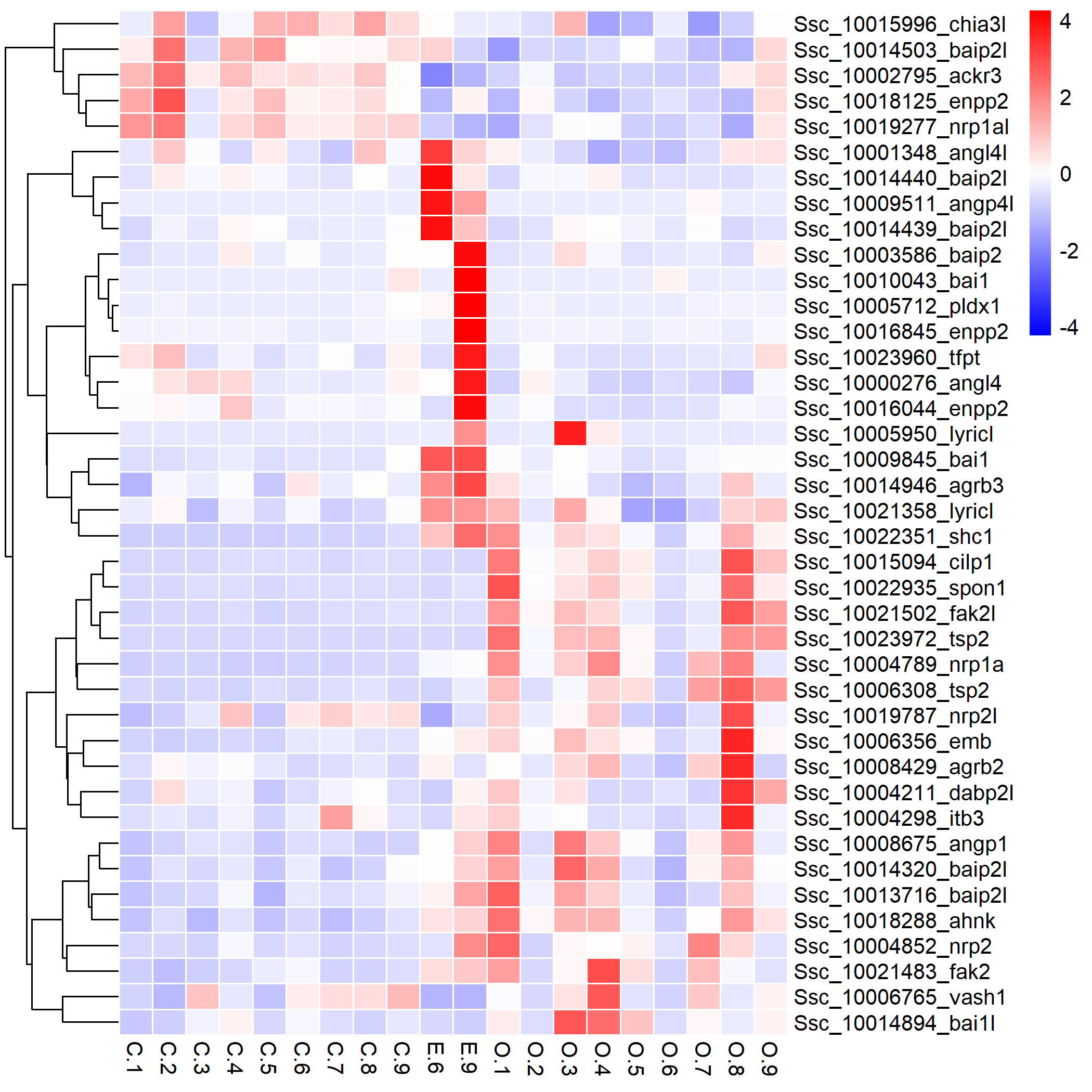


**Fig. S5** Expression pattern of genes involved in regulation of angiogenesis in the ovary in the reproductive cycle. A) Heatmap was constructed by comparing three kinds of samples at different stage of the reproductive cycle. The x‐axis shows sampled tissues with the prefix C. for connective tissue rich in blood vessels covering the egg membrane and E. for embryos and O. for ovarian wall. Arabic numerals represent different stages. 1, pre-fertilization; 2, 1-cell; 3, 8-cells; 4, 16-cells; 5, gastrula stage; 6, 8-somites stage; 7, tailbud stage; 8, pre-hatching; 9, hatching.
