# Supplementary material for: The Bdkrb2 gene family provides a novel view of viviparity adaptation in Sebastes schlegelii
Source: BMC Ecol Evol. 2021 Mar 17;21:44. doi: 10.1186/s12862-021-01774-0 (PMC7968187; doi:10.1186/s12862-021-01774-0)
Supplement: Supplementary file 8 — Additional file 8: Table S2. Sample and data statistics of ovarian RNA-seq. [file 12862_2021_1774_MOESM8_ESM.docx]

**Table. S2 Sample and data statistics of ovarian RNA-seq**

| **Sample stage** | **Sample name** | **RIN value** | **Raw** | **Clean** | **Clean** | **Clean** | **Clean** | **Clean** |
| --- | --- | --- | --- | --- | --- | --- | --- | --- |
|  |  |  | **reads** | **reads** | **bases** | **Reads** | **Reads** | **Reads** |
|  |  |  | **(M)** | **(M)** | **(Gb)** | **Q20 (%)** | **Q30 (%)** | **Ratio (%)** |
| pre-fertilization | C-1 | 5.90 | 165.50 | 153.13 | 15.31 | 98.80 | 93.96 | 92.53 |
| 1-cell | C-2 | 6.30 | 191.94 | 178.85 | 17.89 | 98.55 | 93.27 | 93.18 |
| 8-cell | C-3 | 6.50 | 174.35 | 162.29 | 16.23 | 98.65 | 93.53 | 93.08 |
| 16-cell | C-4 | 7.20 | 224.59 | 210.96 | 21.10 | 98.70 | 93.74 | 93.93 |
| gastrula | C-5 | 6.60 | 179.39 | 167.41 | 16.74 | 98.58 | 93.32 | 93.32 |
| 8-somites | C-6-1 | 6.40 | 184.13 | 170.23 | 17.02 | 98.50 | 93.30 | 92.45 |
| 8-somites | C-6-2 | 6.40 | 157.99 | 145.72 | 14.57 | 98.42 | 92.99 | 92.24 |
| tailbud | C-7 | 5.90 | 138.37 | 128.33 | 12.83 | 98.82 | 94.09 | 92.74 |
| pre-hatching | C-8 | 6.50 | 206.55 | 190.83 | 19.08 | 98.47 | 93.24 | 92.39 |
| hatching | C-9-1 | 6.10 | 140.75 | 129.74 | 12.97 | 98.45 | 93.15 | 92.18 |
| hatching | C-9-2 | 4.60 | 119.62 | 116.26 | 11.63 | 98.62 | 95.37 | 97.19 |
| hatching | C-9-3 | 7.70 | 139.61 | 129.42 | 12.94 | 98.55 | 93.48 | 92.70 |
| pre-fertilization | O-1 | 5.90 | 160.84 | 150.29 | 15.03 | 98.89 | 94.39 | 93.44 |
| 1-cell | O-2 | 6.20 | 176.77 | 164.35 | 16.44 | 98.61 | 93.75 | 92.98 |
| 8-cell | O-3 | 5.60 | 147.72 | 136.31 | 13.63 | 98.81 | 94.03 | 92.28 |
| 16-cell | O-4 | 5.50 | 213.76 | 199.28 | 19.93 | 98.93 | 94.53 | 93.23 |
| gastrula | O-5 | 5.00 | 169.32 | 157.59 | 15.76 | 98.79 | 94.01 | 93.08 |
| 8-somites | O-6-1 | 5.00 | 209.14 | 195.22 | 19.52 | 98.75 | 93.78 | 93.35 |
| 8-somites | O-6-2 | 2.20 | 161.60 | 156.76 | 15.68 | 98.66 | 95.60 | 97.01 |
| tailbud | O-7 | 6.90 | 170.91 | 158.91 | 15.89 | 98.64 | 93.78 | 92.98 |

Sample name showed tissues with the prefix. C. represented connective tissue rich in blood vessels covering the egg membrane and E. indicated embryos and O. represented ovarian wall.
